# Incident gout and weight change patterns: a retrospective cohort study of US adults

**DOI:** 10.1186/s13075-021-02461-7

**Published:** 2021-03-02

**Authors:** Lu Bai, Jian-Bo Zhou, Tao Zhou, Roger B. Newson, Marly Augusto Cardoso

**Affiliations:** 1 Hospice & Palliative Care Unit, Haidian Section of Peking University Third Hospital, Beijing, China; 2 Department of Endocrinology, Beijing Tongren Hospital, Capital Medical University, Beijing, China; 3 School of Public Health (Shenzhen), Sun Yat-sen University, Guangzhou, 510006 Guangdong China; 4 Department of Primary Care and Public Health, Imperial College London, London, UK; 5 Department of Nutrition, School of Public Health, University of Sao Paulo, Sao Paulo, Brazil

**Keywords:** Gout, Weight change, Cohort

## Abstract

**Background:**

Although the relationship between obesity and incident gout has been clarified, the influence of weight changes during the transition from early adulthood to midlife and the different weight change patterns in specific age ranges on the incidence of gout in later life remain unknown. Therefore, we aimed to investigate the association between weight change patterns across adulthood and incident gout.

**Methods:**

Using data from the National Health and Nutrition Examination Survey (NHANES), we categorized individuals into four weight change patterns: those who remained obese (stable obese), those who moved from a non-obese body mass index (BMI) to an obese BMI (gaining), those who moved from an obese BMI to a non-obese BMI (losing), and those who remained non-obese (stable non-obese). Incident gout reflected its occurrence over the 10-year follow-up from the recalled midlife weight measure to the time of this survey. Hazard ratios (HRs) and 95% confidence intervals relating weight change patterns to incident gout over the 10-year follow-up period were calculated using Cox models adjusted for covariates. The hypothetical population attributable fraction (PAF) for the weight change patterns was calculated.

**Results:**

Among our sample of adults aged 40–74 years at their midlife weight measure (*n* = 11,079), 320 developed gout. The highest risk of incident gout was found for participants with the stable obese pattern (HR 1.84; 1.08–3.14) and not for participants who remained stable non-obese during adulthood. Moreover, gaining weight was a significant risk factor for incident gout (HR 1.65; 1.19–2.29). No significant associations were found between losing weight change patterns and the risk of gout during the study period. If participants who gained weight had become non-obese during the 10-year follow-up, an estimated 3.2% (95% CI 0–6.3) of observed gout cases could have been averted. In addition, if the population had maintained a normal BMI, 32.9% (95% CI 18.2–44.9) cases could have been prevented during the 10 years.

**Conclusions:**

Gaining weight over adulthood was associated with an increased risk of gout. These findings have highlighted that maintaining non-obese weight and weight loss across adulthood is essential for the prevention and treatment of gout in adult life.

## Background

Gout is a common form of inflammatory arthritis [1], with the overall prevalence of gout among US adults at 3.9% [2]. Characterized by monosodium urate (MSU) crystal deposition resulting from chronic elevation of serum uric acid (SUA) levels [3, 4], gout can lead to severe arthropathies, physical impairment, and a decreased quality of life [5]. The general management principle is to reduce SUA levels, allowing MSU crystals to dissolve, leading to the elimination of acute attacks, and possibly curing the disease [6–8]. A large epidemiological study has indicated that the worldwide prevalence of gout is on an upward trend, with increasing obesity being a significant risk factor for gout development [9]. Gout is also affected by numerous factors, including intake of sugar-sweetened beverages (SSB), alcohol consumption, renal disease, and the use of diuretics and antihypertensive drugs [10–12]. A recently published meta-analysis indicated that SSB consumption was significantly associated with an increased risk of gout in the adult population [13]. Obesity is also a major global health challenge [14]. The association between obesity and gout may be attributed to insulin resistance, which in turn reduces renal urate excretion, resulting in hyperuricemia [15]. The American College of Rheumatology guidelines recommend weight loss as part of gout management for patients with obesity [16].

Although some prospective studies have clarified the relationship between obesity and the incidence of gout [17–21], the influence of weight changes during the transition from early adulthood to midlife and the different weight change patterns in specific age ranges on the incidence of gout in later life remain unknown. Hence, assessment of the population-level effect of weight change across the life course on incident gout risk is needed.

We test a hypothesis that individuals who were losing weight had a higher risk of gout than individuals who maintained a non-obese BMI over time (“residual risk” hypothesis), which means that individuals who have been obese are at higher risk of developing gout than those who have never been obese. Therefore, we aimed to investigate the association between weight change patterns across adulthood and incident gout.

## Methods

### Data sources

**Fig. 1:**
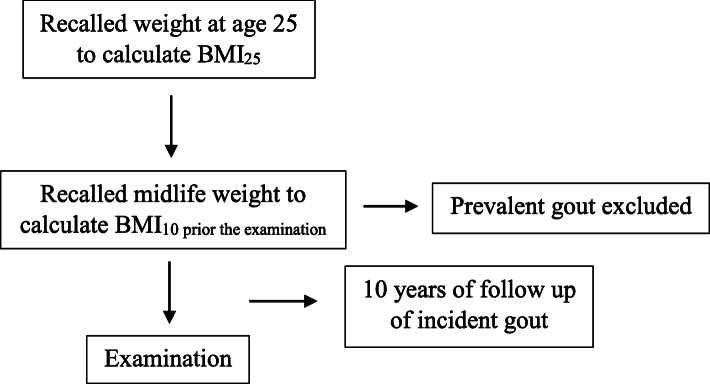
Study design for analysis of incident gout (*n* = 11,079). Creation of a cross-sectional, retrospective cohort of US adults. We studied individuals who participated in the NHANES (2007–2014) cross-sectional survey at ages 40–74 years. It was based on the participants’ memory recalled weight history at age 25 (young adulthood) and 10 years before the survey (age 30–64 years, midlife), which was used to create a measure of weight change between young adulthood and midlife. We then investigated the association between this weight change and the successive risk of developing gout. “Follow-up” for incident gout began 10 years before the survey. Individuals who reported receiving a first diagnosis of gout between 10 years before the survey and time of the survey were considered to have incident gout

This was a retrospective longitudinal study using national data from the National Health and Nutrition Examination Survey (NHANES) (2007–2014), which is a nationally representative sample of US adults. The survey contains information on graphic characteristic, weight history, and health behaviors/conditions [22]. We used the data of adults aged 40–74 years who participated in NHANES during this period. Self-reported weight change was assessed based on the participants’ memory recall of weight at age 25 and 10 years prior to participation in NHANES. Gout status was determined by a self-report of a prior diagnosis, and age at diagnosis was used to establish the time of gout onset. Therefore, we investigated the association between weight change and the risk of incident gout over the 10 years from the midlife measure to the time of the survey. The study design depicted in Fig. 1 was modified from a similar analysis of incident diabetes [23]. Institutional Review Board approval was not required for this study because the investigation was based on secondary analyses of publicly available, deidentified data.

#### Weight change measures

Participants were requested to recall their weight at age 25 and 10 years prior to participation in NHANES. In this study, BMI at age 25 was calculated using the reported height at age 25, which we considered as young adulthood. Likewise, we regarded the recalled weight 10 years prior to the survey as a measure of midlife weight. Owing to the possibility of declining height with age, the reported height at age 25 was used. The height measured in the survey was used to calculate the BMI at midlife. BMI at age 25 (BMI_25_) and 10 years prior to the survey (BMI_10prior)_ was calculated as weight (kg) divided by the square of height (m^2^). BMI change patterns, according to the BMI change trajectory were categorized into four groups from BMI_25_ to BMI_10 prior_. The groups were as follows [1]: stable non-obese, BMI_25_ < 30 kg/m^2^ and BMI_10 prior_ < 30 kg/m^2^; losing, BMI_25_ ≥ 30 kg/m^2^ and BMI_10 prior_ < 30 kg/m^2^; gaining, BMI_25_ < 30 kg/m^2^ and BMI_10 prior_ ≥ 30 kg/m^2^; and stable obese, BMI_25_ ≥ 30 kg/m^2^ and BMI_10 prior_ ≥ 30 kg/m^2^.

#### Assessment of incident gout

Participants were considered to have gout if they reported in the survey that they had been diagnosed by a health care provider as having gout (gout incidence during follow-up was included based on the information on gout diagnosis and the use of gout medications).

#### Statistical analysis

Tests of statistical significance were two-tailed, with a significance level of 0.05. STATA15.0 (StataCorp LLC, College Station, TX, USA) was used for all analyses. All analyses were performed using SUDAAN (SUDAAN Language Manual, Release 9.0, 2004; Research Triangle Institute, NC, USA), which considered the complex sampling design used in NHANES, thus yielding unbiased standard error estimates [24]. The percentages of missing values of covariates were less than 5%, except for income (6.8%) [24]. We compared the baseline characteristics by weight change patterns using the *χ*^2^ test for categorical variables and an analysis of variance adjusted for sampling weights for continuous variables.

Our hypothesis that individuals who were obese or gained weight had a higher risk of gout than individuals who maintained a non-obese BMI over time (“residual risk” hypothesis) was tested using Cox models to predict the rate of incident gout across the four BMI change groups over the 10 years. Stable non-obese weight change patterns were used as the reference category against which all the other weight change patterns were compared. The models included adjustments for ethnicity, sex, age, current smoking (yes/no), annual household income, diabetes, hypertension, and consuming alcohol.

#### Hypothetical scenarios

Estimates of the percentage of gout cases that could be averted under three hypothetical scenarios were calculated using the following equation for the population attributable fraction (PAF):

$$ PAF=\sum \limits_{i=0}^k{pd}_i\left(\frac{HR_i-1}{HR_i}\right) $$

where *pd*_*i*_ was the proportion of total incident cases in the sample observed in the *i*th weight change category and *HR*_*i*_ was the hazard ratio associated with that category [25]. We estimated the fraction of cases that would be eliminated if a weight change category was redistributed to another category. To allow for a valid calculation of PAF, logistic regression models with covariates were included in the model. For these analyses, we used a single binary categorical variable and compared participants in the weight change category with the rest of the population to calculate the PAF in the total population for the relevant individuals [26].

In the first scenario, we estimated what would have happened if those who were gaining weight had a non-obese BMI at age 25 and during midlife. In the second scenario, we calculated the proportion of averted cases if those who had a stable obese BMI during the period had a non-obese weight. In the third scenario, we examined the percentage of the entire population if participants had a normal BMI at age 25 and during midlife.

## Results

### Clinical features of the participants

The clinical characteristics of the participants according to the weight change category are shown in Table 1. Overall, 11,079 participants were included in the study, and 320 cases of incident gout were identified during the 10-year follow-up. More than half of the sample (78.2%) maintained their BMI category. In specific, 7945 (71.7%) participants were in the stable non-obese group; losing group was rare, with 115 (1.0%); 2307 (20.8%) were gaining group; and 712 (6.5%) were stable obese group.

**Table 1:** Characteristics at survey of weight change patterns from age 25 to 10 years prior to the survey in adults ages 40–74 years* (*N* = 11,079)

	Stable non-obese	Losing body weight	Gaining body weight	Stable obese
No of participates, *n* (%)	7945 (71.7)	115 (1.0)	2307 (20.8)	712 (6.5)
Age (years), (95% CI)	54.1 (53.7–54.4)	52.0 (50.6–53.5)	56.8 (56.2–57.4)	51.3 (50.4–52.1)
Female, *n* (%)	4082 (53.0)	49 (45.5)	1198 (49.9)	368 (46.6)
Black, *n* (%)	1699 (9.9)	27 (16.2)	578 (11.6)	239 (17.3)
Current smoke, *n* (%)	1799 (21.1)	39 (34.9)	386 (16.4)	150 (18.3)
Alcohol consumption (drinks/day), (95% CI)	1.6 (1.6–1.7)	2.1 (1.5–2.8)	1.4 (1.3–1.5)	1.7 (1.4–2.0)
Diabetes, *n* (%)	793 (6.8)	22 (15.8)	684 (24.6)	217 (25.9)
Hypertension, *n* (%)	2978 (33.2)	48 (41.8)	1392 (57.1)	419 (56.5)
Annual household income ≥ 65,000$, *n* (%)	2644 (47.8)	29 (32.1)	671 (41.4)	187 (38.4)
BMI, mean (95% CI), kg/m^2^				
At 25 years of age^a^	22.1 (22.0–22.2)	34.5 (33.1–35.9)	24.9 (24.7–25.1)	34.7 (34.1–35.2)
At 10 years prior^b^	24.6 (24.5–24.7)	26.7 (26.0–27.4)	34.2 (34.0–34.5)	39.0 (38.2–39.9)
At examination^c^	26.9 (26.7–27.0)	30.5 (28.9–32.1)	34.8 (34.5–35.1)	39.3 (38.5–40.2)
Serum uric acid (mg/dL)	5.30 (5.25–5.35)	5.65 (5.23–6.08)	5.82 (5.73–5.90)	5.93 (5.77–6.10)

*NHANES (2007–2014); sample weighted estimates

^a^Self-reported BMI at 25 years of age

^b^Self-reported BMI 10 years before examination

^c^Self-reported BMI at examination

Different weight change patterns showed a different prevalence of comorbidities, such as diabetes mellitus (6.8%, stable non-obese group; 15.8%, losing group; 24.6%, gaining group; and 25.9%, the stable obese group) and hypertension (33.2%, stable non-obese group; 41.8%, losing group; 57.1%, gaining group; and 56.5%, stable obese group).

### Association of weight change patterns and incident gout

Compared with that of stable non-obese participants, stable obese participants had a higher risk (1.84; 95% CI 1.08–3.14) of developing gout. Those who gained weight from young adulthood through midlife had 1.65 times (95% CI 1.19–2.29) the risk of developing gout during the 10-year follow-up (Table 2). No significant difference (HR 0.48; 95% CI 0.09–2.61) was observed in the risk of gout onset between those reporting a shift from an obese BMI to a non-obese BMI over the study period.

**Table 2:** Residual risk: HRs for weight change and incident gout, NHANES (2007–2014) (*n* = 11,079)

Obesity track	HR^†^	95% confidence interval	*P* value
Stable obese	1.84	(1.08–3.14)	0.02
Gaining weight	1.65	(1.19–2.29)	0.003
Losing weight	0.48	(0.09–2.61)	0.39
Stable non-obese	Reference	1.0	

*NHANES* National Health and Nutrition Examination Survey (2007–2014)

^†^Adjusted for ethnicity, sex, age, current smoke (yes/no), annual household income, diabetes, hypertension, and alcohol consumption

### Cumulative incident gout defined by the onset in the recent 10 years according to the obesity track

**Fig. 2:**
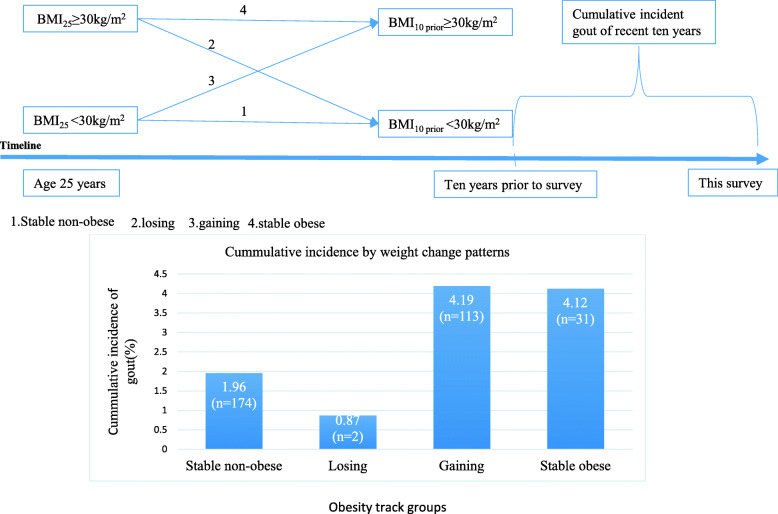
Cumulative incident gout of recent 10 years by weight change category from age 25 to 10 years before the survey. (1) Stable non-obese, BMI_25_ < 30 kg/m^2^ and BMI_10 prior_ < 30 kg/m^2^; (2) losing, BMI_25_ ≥ 30 kg/m^2^ and BMI_10 prior_ < 30 kg/m^2^; (3) gaining, BMI_25_ < 30 kg/m^2^ and BMI_10 prior_ ≥ 30 kg/m^2^; and (4) stable obese, BMI_25_ ≥ 30 kg/m^2^ and BMI_10 prior_ ≥ 30 kg/m^2^

Figure 2 illustrates the cumulative incidence curves by time for each weight change group. The cumulative incidence of onset gout was 1.96% (95% CI, 1.58–2.43) for sustained non-obese, 0.87% (95% CI, 0.15–4.74) for loss, 4.19% (95% CI, 3.31–5.28) for gain, and 4.12% (95% CI, 2.53–6.63) for stable obesity.

#### Hypothetical scenarios

The three hypothetical scenarios that were defined earlier were used to calculate the PAFs (Table 3). In the first scenario, if those who were gaining weight but had a non-obese BMI, 11.5% (95% CI 4.6–17.9) of observed gout cases could have been averted. In the second scenario, if those who maintained an obese BMI during the period had a non-obese weight, 3.2% (95% CI 0–6.3) of the observed cases could have been averted. In the third scenario, maintaining a normal BMI between young adulthood and midlife of the total population could have prevented 32.9% (95% CI 18.2–44.9) of gout cases.

**Table 3:** Population attributable fractions (PAF) for population counterfactuals of incident gout among weight change patterns across adulthood

Scenario^†^	PAF (%), 95% CI total population	PAF (%), 95% CI subpopulation^#^
1. From gaining to stable non-obese	11.5 (4.6–17.9)	36.4 (18.6–50.3)
2. From stable obese to stable non-obese	3.2 (0–6.3)	37.6 (6.0–58.7)
3. Total population to stable normal	32.9 (18.2–44.9)	42.8 (25.8–55.9)

Source: NHANES (2007–2014)

^†^Adjusted for ethnicity, sex, age, current smoke (yes/no), annual household income, diabetes, hypertension, and alcohol consumption

From a younger age to an older age: scenario 1, if those who gained weight, instead remained non-obese; scenario 2, if those who maintained an obese BMI, instead lost to a non-obese BMI; scenario 3, if the total population had a normal BMI from young adulthood that was maintained through midlife

^#^Scenario 1: subpopulation represents the gaining group; scenario 2: subpopulation represents the stable obese group; scenario 3: subpopulation represents the population whose BMI ≥ 25 kg/m^2^

## Discussion

Our objective was to evaluate the relationship between obesity and weight change and the incidence of gout in a large retrospective study of nationally representative US adults. We found that participants with a stable obese BMI gaining weight to shift from a non-obese to an obese group were consistently associated with the risk of gout. These results also emphasized the importance of maintaining non-obese weight across adulthood, especially weight loss for obese individuals, for reducing gout risk in later life.

### Comparisons between previous studies and our results

The risk of incident gout owing to the increased BMI was substantial. In our cohort, participants with an obese BMI and who had a stable obese BMI had a high possibility of developing gout. Since the prevalence of obesity continues to rise, having reached 37.7% according to NHANES [27], the absolute excess risk of gout due to excess weight is also expected to increase. These results expanded on the findings of previous studies on weight change and gout. A study conducted in China had found that those who had a weight gain of 5 kg, 20 kg, or higher from early to midlife were associated with a higher risk of gout, regardless of sex [28]. Furthermore, a systematic review and meta-analysis had found that in obese individuals, gout was 2.24 times more likely to occur than in those with normal weight [29]. A population-based cohort study had also indicated that obese individuals have an adjusted 2.4-fold higher risk of developing gout than non-obese individuals during a 9-year follow-up [20]. The Health Professionals Follow-up Study (HPFS) [30] prospectively evaluated the relations between BMI, weight change, and incident gout. It had found that weight gain since young adulthood was strongly associated with the risk of gout, even after adjusting for age, hypertension, alcohol consumption, and diet. These findings supported the results from our study that the HRs of gout were 1.65 for gaining individuals and 1.84 for stable obese individuals during the 10-year period. Studies have also found that the cumulative incidence of gout at age 70 was 11.8% with BMI ≥ 35 kg/m^2^, but it was only 1.9% in those with BMI < 25 kg/m [29, 31]. Similar results were observed in our study stratified by weight change patterns from age 25 to 10 years prior to the survey, with the following cumulative incidence curves by time for each weight change group: 1.96% (1.58–2.43) for stable non-obese, 0.87% (0.15–4.74) for losing, 4.19% (3.31–5.28) for gaining, and 4.12% (2.53–6.63) for stable obesity. The potential impact of weight gain on the incidence of gout was substantial.

Nonetheless, we found that weight loss was associated with a reduced risk of gout. Likewise, gout patients losing weight through bariatric surgery or diet experienced reduced flare frequency [32]. A meta-analysis of prospective studies had suggested that obesity was associated with a dose-dependent increase in the relative risks (RRs) of incident gout and that gout incidence could have been lessened by interventions aimed at obesity [33]. The hypothetical scenarios targeted to explore the potential effect of weight gain and weight loss interventions.

In the hypothetical scenario preventing weight gain in the population after age 25 and maintaining a non-obese BMI, an 11.5% (95% CI 4.6–17.9) reduction in gout cases in the population could have occurred. If all those who were obese at age 25 lost to a non-obese BMI by midlife, 3.2% (95% CI 0–6.3) of observed incident gout could have been averted. Likewise, we found that 32.9% (95% CI 18.2–44.9) of gout cases during this period could have been averted if all individuals in the population maintained a normal weight from early adulthood to midlife. Our hypothetical scenarios were consistent with previous studies that examined the effect of weight loss on gout.

Studies on the effects of weight loss interventions from either bariatric surgery or lifestyle modification (moderate calorie/carbohydrate restriction, increased proportional intake of protein and unsaturated fat) also reported a reduced incidence of gout [32, 34, 35]. A systematic review [36] shows that a weight loss of > 3.5 kg showed beneficial effects on gout attacks at medium-term/long-term follow-up. However, in our study, the losing group had a wide 95% CI because weight loss from an obese BMI to a non-obese BMI was rare, accounting for only 1.0% of the total population.

### Potential mechanisms

In some studies [37, 38], weight gain has been associated with increasing SUA levels. Conversely, weight loss reduced the level of SUA [39, 40]. A study showed that a higher BMI increased the risk of gout by increasing the SUA level [30], with high SUA levels resulting in the formation of MSU crystals [41], which then contributed to an inflammatory response with tissue damage [42, 43]. Obesity possibly increased the production of MSU crystals and reduced renal excretion of urate [44, 45], causing hyperuricemia. Although most people with hyperuricemia do not have gout, increased SUA levels can still raise the risk of developing gout [46]. Several studies have demonstrated a strong association among gout, SUA levels, obesity, and metabolic syndrome, including hypertension and dyslipidemia [22, 47, 48]. The study by Lyndgoh et al. [49] revealed that raised SUA levels were an outcome but not a cause of obesity, which had been assumed to lead to high SUA levels causing impaired nitric oxide production, endothelial dysfunction, enhanced oxidative stress, and maladaptive immune and inflammatory responses [50].

In 2020, the ACR Guideline for the Management of Gout [51] was conditionally recommended in areas such as diet (alcohol intake, vitamin C supplement, and high-fructose intake), lifestyle, or concomitant medications, which might have affected the SUA levels in gout patients. The NRHS [52] cohort showed a 51% reduction in the risk of gout with a high intake of fruit, whereas the HPFS [53] cohort showed a 63% increased risk. The results of the two studies were conflicting, and we assumed that while the HPFS cohort [53] included fruit with high-fructose content, the NRHS [52] cohort included fruit that may have represented a healthier dietary intake. In short-term feeding trials and experimental studies, high-fructose intake resulted in hyperuricemia and incident gout and increased insulin resistance [53, 54]. In NHANES, artificially sweetened carbonated beverage consumption was associated with higher SUA levels [55]. In addition, a cross-sectional study has suggested that cereal and yogurt consumption may have been associated with a lower SUA level [56]. Urate-lowering therapy was highly recommended in the 2020 ACR Guideline for the Management of Gout, which had high quality of evidence supporting its use as an efficient treatment in reducing flare frequency and SUA levels [57–59].

### Strengths and limitation

The strengths of our study are as follows: first, the use of a retrospective cohort design allowed us to take advantage of a large, nationally representative cohort of US adults to estimate associations between weight change and incident gout from early adulthood to midlife. Therefore, our estimates are broadly generalizable to the US population. Second, we tested a hypothesis concerning the residual risk associated with previously having a higher weight.

This study also had several limitations. First, weight at early adulthood was recalled at a later age, and some misclassifications of weight change were inevitable. However, evidence has demonstrated that historic weight measures can have high agreement with the measured weight [60, 61]. Second, although spurious estimates of BMI may have been made when relying on self-reported height at age 25 in adults older than age 50 to calculate BMI at age 25 [62], evidence has suggested a relatively high level of accordance between height recall and historically measured height among older adults [63, 64]. Third, the definition of gout in epidemiological studies varies and includes, either in isolation or combination, physician-diagnosed gout based on history and examination, prescription practices, and analysis of healthcare datasets or coding systems. We believe that the self-reporting of medically diagnosed gout is the real definition for epidemiological purposes, which is supported by a 2011 US study that explicitly addresses the question of reliability and sensitivity of self-reported physician-diagnosed gout [65]. Using a large cohort consisting of more than 32,000 participants, this group found that self-reported physician-diagnosed gout was consistent over multiple questionnaires and the duration of time. Self-reported of physician-diagnosed gout had a sensitivity of 84% while compared with that of the gold standard, which was defined as a hospital discharge diagnosis of gout or use of gout-specific medications (colchicine, probenecid, allopurinol).

## Conclusions

The NHANES survey data were analyzed using a novel application to explore the relationship between weight change patterns and incident gout through early adulthood to midlife. Regarding the residual risk hypothesis, those who were stable obese had a higher risk of incident gout than those who remained non-obese throughout the life span. Our hypothetical scenarios indicated beneficial effects of positive weight intervention change from either stable obese or gaining weight to sustained normal or from stable obese to weight loss. Taken together, the findings support that maintaining normal weight over the whole of adulthood is beneficial reduces the risk of gout among obese individuals. The significance of developing policies and programs that reduce the prevalence of obesity is underscored by the results of our study. Identifying populations at risk of developing gout may provide opportunities for primary prevention. Clinical trials on long-term health consequences of weight intervention are warranted.

## Data Availability

Data from the National Health and Nutritional Examination Survey are available online through the CDC.

## Abbreviations

BMI: Body mass index
SUA: Serum uric acid
PAF: Population attributable fraction
HR: Hazard ratios
NHANES: National Health and Nutritional Examination Survey
